# An Age-Related Morphometric Profile of Skeletal Muscle in Healthy Untrained Women

**DOI:** 10.3390/jcm5110097

**Published:** 2016-11-07

**Authors:** Anastasia Bougea, George Papadimas, Constantinos Papadopoulos, George P. Paraskevas, Nikolaos Kalfakis, Panagiota Manta, Evangelia Kararizou

**Affiliations:** First Department of Neurology, Eginition Hospital, School of Medicine, National and Kapodistrian University of Athens, Vass. Sofias Ave. 72–74, Athens 11528, Greece; gpapad@yahoo.gr (G.P.); constantinospapadopoulos@yahoo.gr (C.P.); geoprskvs44@gmail.com (G.P.P.); nikalfak@med.uoa.gr (N.K.); pmanta@med.uoa.gr (P.M.); ekararizou@med.uoa.gr (E.K.)

**Keywords:** morphometry, histochemistry, muscle biopsy, fiber type, aging

## Abstract

There is a paucity of data on muscle biopsies in females of mixed ages in terms of age-related changes. Cross sections of autopsy material including the quadriceps femoris and biceps brachii muscles were obtained from 23 healthy women, aged 24–82 years, who had suffered sudden death. We calculated the percentage of the number, and the mean diameter, of type I and type II muscle fibers within the fascicles as well as in their peripheral parts. The number of type II fibers were shown to reduce significantly with age (*p* < 0.005), especially in the fascicle periphery, but the percentage of type 1 fibers did not alter significantly. It was noted that type II fibers diminished in size with age, indicating a relationship between fiber size and age. This result became more apparent in the fascicle periphery (*p* < 0.05). In women, type II muscle fibers were seen to reduce in size and number with advancing age. We postulate that regular physical activity can increase the size of type II muscle fibers, thus helping to both prevent and treat age-related muscle loss.

## 1. Introduction

Generally, women have smaller and weaker muscles than men [1]. Senescence has been linked to both loss of muscle mass and strength, which varies according to gender and muscle status activity. The decline of muscle mass is the result of a considerably lower number of type I and type II muscle fibers, as well as reduced muscle cell size, and favored atrophy of type II fibers [2]. Both type I and type II adult muscle fibers are larger in men than women. In men, type II muscle fibers are usually larger than type I fibers, whereas the opposite occurs in women. The impact of a long period of physical activity on age-related modifications in skeletal muscle structure has been studied; however, data on gender-related morphological changes are lacking. Loss of type II fibers has been associated with physical inactivity, mainly in men [3,4,5,6,7], which can be prevented by endurance training [8]. An understanding of the extent to which aging and physical activity processes induce muscle fiber loss has not been elucidated.

As far as we know, this is the first attempt to examine the association of age and long periods without physical exercise with the morphometric features of healthy skeletal muscle in women of different ages. It is important to realize how the aging neuromuscular system in females adjusts to physical activity. The purpose of this brief report was to define the extent, if any, of age association with quantitative changes in fiber size, their structure, and arrangement of both fiber types within and on the periphery of the muscle fascicle in healthy untrained women.

## 2. Experimental Section

We designed a cross-sectional study based on specimens from the quadriceps femoris and biceps branchii from adult cadavers of 23 healthy women that were separated into two groups. Group A included 11 females under the age of 41 years, and Group B included 12 females over the age of 41. No history of neuromuscular or other disorders was recorded that could directly or indirectly affect the peripheral nervous system or skeletal muscle structure. All subjects were healthy, without participating in any exercise training program for the past five years. Their anthropometric characteristics are outlined in Table 1.

This study was performed according to Greek law, which allows the use of cadavers for research in universities. Muscle samples were taken from the middle portion of the quadriceps (vastus lateralis) of the right leg using surgical biopsy procedures. Fascicles were randomly selected from the muscle sections. Using a cryostat (Leica CM 3000) at −24 °C, serial 10 m transverse sections were cut and mounted on glass slides. The sections were then stained for a myofibrillar ATPase (pH 9.4) according to the modified protocol [9]. It was demonstrated that two fiber types can be defined in autopsy material [10]. An automatic image analyzer (Image-Pro Plus, version e4, 5.1-Media Cybernetic) was applied for morphometric analysis with the following measurements: (a) the number of type I and II muscle fibers; (b) the diameter of both types; (c) the percentage of the number, and the mean diameter, of the two types in the interior and in the peripheral area of the fascicles. An average minimum of 400 fibers per section was analyzed. We used the smallest fiber diameter to calculated the size of the fibers [10]. The results for each muscle were appraised according to age group. A Student’s *t*-test and a linear regression, along with Spearman’s non-parametric method and an analysis of variance (2-way ANOVA) were applied for statistical analysis. A non-parametric Mann–Whitney U test was used to compare the anthropometric data between two groups. Power analysis showed that a sample size of 23 subjects would provide 80% power, at a level of *p* = 0.05 [11]. The Ethics Committee of Eginition Hospital approved this study and provided written informed consent in accordance with the Declaration of Helsinki.

## 3. Results

In both biceps and quadriceps, we found a significant reduction in the number of type II fibers with age (*p* < 0.005), while this association was not significant for type I fibers (Table 2).

The elderly group displayed a significantly higher percentage (*p* < 0.001) of type II fibers in the fascicle periphery. The difference in percentage between internal and peripheral type I fibers was 4.2. The mean diameter of both types was less in the fascicle periphery as regards both biceps and quadriceps, but the difference was not statistically significant (Table 3). With age, type II fibers reduced in size, both in the peripheral and the internal part of the fascicle, thus indicating an association between fiber size and age. This result became more obvious in the fascicle periphery (*p* < 0.05) (Table 3).

## 4. Discussion

This report explores age-associated discrepancies in morphometric characteristics of healthy untrained females. The main findings were as follows: (i) a progressive decline in type II fibers with advancing age was noted, both in terms of size and number; (ii) reduced muscle fiber diameter was more apparent in the fascicle periphery.

Gender-related morphological differences, though debatable, have also been noted in terms of fiber size within the quadriceps. So far, most studies have shown that constant endurance training does not delay waste of muscle mass and atrophy of type II fibers associated with the aging process [8,12,13,14]. However, a study conducted by Klitgaard et al. 1990 found that muscle fiber size and muscle force characteristics among elderly men with 12–17 years of heavy resistance training were comparable to those of young adult control subjects.

Published data regarding the overall number of fast and slow fibers in the vastus lateralis muscle of men vs. women are contradictory. Some studies have found a significantly higher percentage of type I fibers in women [15,16], some reported a higher percentage of type I fibers in men [17,18], while others found no difference in terms of gender [19]. Such disparity could be attributed to differences in sample size, age, methodology, hormone profiles, and physical activity level. Notably, all subjects were middle-aged and untrained.

Our results were comparable with those of several cross-sectional studies that demonstrated a reduced number and mean area of type II muscle-fast fibers in untrained [3,4,5,6,7] as well as in endurance-trained subjects [8,13,14]. A plausible explanation could be that reduced resistance training among older subjects could arguably have contributed to the decrease in fast fiber size, although selective involvement of motor neurons, particularly the larger motor neurons innervating type II fibers, cannot be ruled out [20]. Contrary to the above studies focused mainly in men, evidence of the relationship between the temporal atrophy of type II fibers in younger women and that in elderly women is lacking. To our knowledge, this is the first study focusing on a comparison between younger and elderly women.

Despite the “random mosaic pattern”, the observed “grouping” of muscle fibers has also been reported in previous studies [21], but related mechanisms have not been clarified. Fiber type arrangement is the result of a continuous process of combined denervation and partial reinnervation with aging [22]. A higher percentage of type II emerged in our study, but the difference tended to reduce with age, which is in line with the results of Sjöström et al. [23]. A larger number of type II fibers have been described on the surface [21]. This may be a consequence of various parts of the muscle adapting to different functional loads. We cannot exclude an association between the spatial distribution of fiber types and functional properties [24]. There is a connection between fibers of different types, which helps metabolites transfer (for example, lactate). Similar to our findings, perifascicular atrophy was linked to vascular alterations and the age-associated decline of muscle activity [25]. It could be the case that local or other factors contribute to the above organization of the fascicle, but further studies are needed.

Our study has certain methodological limitations, such as its small sample size. Furthermore, the cross-sectional study design does not provide evidence of any relationship between the temporal atrophy of type II fibers in younger women and that in elderly women [26]. Our finding that type II fibers decrease amplitude in elderly females strengthens the hypothesis of type II fiber atrophy in older untrained women, in agreement with other studies focused on men [27]. It is possible that the lack of physical activity in elderly women may be conducive to fast fiber size loss with the aging process. Lastly, the wide age range in our study has the advantage of the emergence of age-associated muscle fiber modifications and power-reproduction features. Our morphometric findings might be important tools for qualitative and quantitative diagnostics of muscle fibers in neuromuscular disorders.

## 5. Conclusions

Untrained women experienced the characteristic age-associated decline in the size of fast fibers previously reported in trained subjects, mostly men. However, mechanisms related to gender differences in aging neuromuscular system remain obscure. In our sample, we demonstrated a considerably larger fiber size compared with those previously reported for untrained older subjects. Physical activity might help to enhance type II muscle fiber size and thus prevent muscle loss with aging in women. Thus, prospective studies should provide recommendations to promote healthy aging.

## Figures and Tables

**Table 1:** Subjects’ anthropometric characteristics.

Subjects’ Group Mean ± SD	Group A (*n* = 11)	Group B (*n* = 12)	*p*-Value
Age, years	31.81 ± 6	65.5 ± 9.35	<0.001 *
Physical height, m	1.71 ± 0.04	1.71 ± 0.05	0.998
Somatic weight, kgr	62.35 ± 5.54	63.5 ± 2.92	0.642
Body fat %	23.64 ± 4.5	23.92 ± 2.87	0.926

PH: physical height; SW: somatic weight mean ± SD standard deviation; * *p* < 0.05 significant.

**Table 2:** Associations of the number of both muscle fiber types within and around the fascicle in the sample of postmortem women.

Women’s Age Range	Around the Fascicle Type Ι Type ΙΙ		*p*-Value		Within the Fascicle Type Ι Type ΙΙ		*p*-Value	
			Quadriceps		femoris			
24–41 yrs	46.4 (1.8)	56.1 (1.6)	n.s. *		50.5 (0.9)	49.3 (1.1)	n.s. *	
45–82 ys	44.7 (1.4)	48.1 (0.9)		0.004 **	48.0 (0.9)	38.2 (0.9)		0.001 **
			Βiceps		branchii			
24–41 yrs	41.9 (1.6)	57.9 (1.9)	n.s. *		60.4 (1.3)	39.5 (1.2)	n.s. *	
45–82 yrs	39.9 (1.3)	46.9 (0.9)		0.002 **	58.3 (1.8)	23.8 (1.1)		0.045 **

Mean (SD-standard deviation), yrs: years, n.s.: non-significant. * *p* for fiber type I and p, ** for type II fibers with age.

**Table 3:** Associations of the mean diameter in μm for both muscle fibers types within and around the fascicle in the sample of postmortem women.

Women’s Age Range	Around the Fascicle Type Ι Type ΙΙ		*p*-Value		Within the Fascicle Type Ι Type ΙΙ		*p*-Value	
			Quadriceps		femoris			
24–41 yrs	42.4 (0.8)	34.0 (0.9)	n.s. *		45.8 (0.8)	38.4 (1.2)	n.s. *	
45–82 yrs	41.7 (0.8)	33.9 (0.9)		0.002 **	44.8 (1.4)	35.8 (1.1)		0.001 **
			Βiceps		branchii			
24–41 yrs	41.3 (1.1)	35.1 (0.8)	n.s. *		45.7 (0.4)	37.3 (2.2)	n.s. *	
45–82 yrs	39.1 (0.5)	34.1 (0.9)		0.002 **	43.8 (1.4)	35.2 (0.8)		0.013 **

Mean (SD-standard deviation), * *p* for fiber type I (periphery) in Group A and Group B, ** *p* for type II fiber (interior) in Group A and Group B, all significant for <0.05. yrs: years, n.s.: non-significant.

## Abbreviations

ATPase	adenosine triphosphatase
PH	physical height
SW	somatic weight
yrs	years
